# Neural stem cells restore myelin in a demyelinating model of Pelizaeus-Merzbacher disease

**DOI:** 10.1093/brain/awaa080

**Published:** 2020-05-18

**Authors:** Fredrik I Gruenenfelder, Mark McLaughlin, Ian R Griffiths, James Garbern, Gemma Thomson, Peter Kuzman, Jennifer A Barrie, Maj-lis McCulloch, Jacques Penderis, Ruth Stassart, Klaus-Armin Nave, Julia M Edgar

**Affiliations:** a1 School of Veterinary Medicine, College of Medical, Veterinary and Life Sciences, University of Glasgow, Glasgow, G12 8TA, UK; a2 Department of Neurology and Center for Molecular Medicine and Genetics, Wayne State University, Detroit, MI 48201, USA; a3 Department of Neuropathology, University Clinic Leipzig, D-04103 Leipzig, Germany; a4 Institute of Infection, Immunity and Inflammation, University of Glasgow, Glasgow, G12 8TA, UK; a5 Max Planck Institute for Experimental Medicine, D-37075 Goettingen, Germany

**Keywords:** leukodystrophy, central nervous system, oligodendrocyte precursor, axon degeneration, dysmyelination

## Abstract

Pelizaeus-Merzbacher disease is a fatal X-linked leukodystrophy caused by mutations in the *PLP1* gene, which is expressed in the CNS by oligodendrocytes. Disease onset, symptoms and mortality span a broad spectrum depending on the nature of the mutation and thus the degree of CNS hypomyelination. In the absence of an effective treatment, direct cell transplantation into the CNS to restore myelin has been tested in animal models of severe forms of the disease with failure of developmental myelination, and more recently, in severely affected patients with early disease onset due to point mutations in the *PLP1* gene, and absence of myelin by MRI. In patients with a *PLP1* duplication mutation, the most common cause of Pelizaeus-Merzbacher disease, the pathology is poorly defined because of a paucity of autopsy material. To address this, we examined two elderly patients with duplication of *PLP1* in whom the overall syndrome, including end-stage pathology, indicated a complex disease involving dysmyelination, demyelination and axonal degeneration. Using the corresponding *Plp1* transgenic mouse model, we then tested the capacity of transplanted neural stem cells to restore myelin in the context of PLP overexpression. Although developmental myelination and axonal coverage by endogenous oligodendrocytes was extensive, as assessed using electron microscopy (*n = *3 at each of four end points) and immunostaining (*n = *3 at each of four end points), wild-type neural precursors, transplanted into the brains of the newborn mutants, were able to effectively compete and replace the defective myelin (*n = *2 at each of four end points). These data demonstrate the potential of neural stem cell therapies to restore normal myelination and protect axons in patients with *PLP1* gene duplication mutation and further, provide proof of principle for the benefits of stem cell transplantation for other fatal leukodystrophies with ‘normal’ developmental myelination.

## Teaser

Gruenenfelder *et al.* report that direct neural stem cell transplantation in an animal model of Pelizaeus-Merzbacher disease restores normal myelin in the face of competition from myelin-competent mutant oligodendrocytes. These data have implications for other fatal leukodystrophies with relatively normal myelin development.

## Introduction

The leukodystrophies comprise a group of genetic disorders that primarily affect the white matter of the CNS (van der Knaap and Bugiani, 2017). Pelizaeus-Merzbacher disease (PMD), the prototypic hypomyelinating leukodystrophy, is caused by mutations in the highly conserved, X-linked *PLP1* gene (Gencic *et al.*, 1989; Boespflug-Tanguy *et al.*, 1994; Garbern *et al.*, 1999), which encodes proteolipid protein (PLP) and its isoform DM20 (Nave *et al.*, 1987). PLP and DM20 are located in compact myelin where they contribute to the normal spacing of the intraperiod line (Boison and Stoffel, 1994; Klugmann *et al.*, 1997; Yool *et al.*, 2002).

Mutations in the *PLP1* gene are associated with a broad spectrum of neurological disorders ranging from severe connatal forms of PMD through intermediate severity, classical forms and *PLP1* null spectrum disorder, to the milder allelic disorder, spastic paraplegia 2 (SPG2) (Garbern and Hobson, 2002; Garbern *et al.*, 2002; Garbern, 2007; Osório and Goldman, 2018). The symptoms of PMD have an early onset and typically include motor and cognitive impairment, nystagmus, choreoathetosis, seizures and ataxia. The underlying pathogenesis of *PLP1* syndromes is distinct for the different genetic abnormalities, which include point mutations, duplication or triplication of the *PLP1* locus and deletion mutation (Woodward, 2008; Torii *et al.*, 2014; Inoue, 2017; Nevin *et al.*, 2017; Osório and Goldman, 2018), partly accounting for the broad clinical spectrum. Modifier genes likely also contribute (Al-Saktawi *et al.*, 2003). Duplications of the *PLP1* locus represent the most common cause of PMD (Sistermans *et al.*, 1998; Mimault *et al.*, 1999); however, variation in the size and break points of the duplicated region on the X chromosome and in the site of recombination likely contribute to the range of severity, even within this genetic subtype (Inoue *et al.*, 1999; Hodes *et al.*, 2000).

To date, no effective treatment is available for PMD, although deferiprone, an iron chelator, reduced oligodendrocyte apoptosis and facilitated myelin formation in the *jimpy* mouse model of connatal PMD (Nobuta *et al.*, 2019), and ketogenic diet (Stumpf *et al.*, 2019), cholesterol supplementation (Saher *et al.*, 2012) and transcriptional suppression by artificial microRNA (Li *et al.*, 2019) have proven beneficial in *Plp1*-overexpressing animal models. Stem cell therapy for PMD has undergone a clinical safety trial in four patients with connatal forms due to point mutations in the *PLP1* gene, in whom myelin is absent by MRI (Gupta *et al.*, 2012, 2019). The question therefore arises whether stem cell therapy might be safe and effective in patients with the most common mutation (*PLP1* duplication), who have, in general, milder pathology and longer life expectancy (Cremers *et al.*, 1987; Sistermans *et al.*, 1998) and in whom the basis for the hypomyelination is likely attributable, at least in part, to loss of previously formed myelin (demyelination) (Inoue *et al.*, 1996; Anderson *et al.*, 1998, 1999; Edgar *et al.*, 2010) and inflammation (Ip *et al.*, 2006; Edgar *et al.*, 2010; Marteyn and Baron-Van, 2016) as suggested from observations in *Plp1*/*PLP1*-overexpressing animal models. This also raises the question whether the exogenous, wild-type oligodendrocytes are able to physically replace myelin made by mutant oligodendrocytes.

Here, we examined two elderly patients with duplication of *PLP1* in whom the overall syndrome, including end-stage pathology, indicated a complex disease involving dysmyelination, demyelination and axonal degeneration. We then systematically characterized myelin development, degeneration and neuroinflammation in a corresponding *Plp1*-transgenic mouse model (*Plp1*-tg mouse, line 72; Readhead *et al.*, 1994) and tested whether cell transplantation directly into the brain could restore normal myelin. We transplanted neural precursors (NPCs) based on earlier observations that murine NPCs form myelin when transplanted into the *Plp1* mutant myelin deficient (*md*) rat (Hammang *et al.*, 1997) and the *Mbp* mutant shiverer mouse (Edgar *et al.*, 2004); a result that was subsequently replicated in shiverer mice using human-derived NPCs (Buchet *et al.*, 2011; Uchida *et al.*, 2012).

We found that transplanted wild-type NPCs were able to differentiate and produce large amounts of compact myelin in *Plp1*-tg mice, against a background of normal-appearing myelination by endogenous mutant oligodendrocytes. This raises the possibility that similar transplantation-based therapy may work in PMD and other leukodystrophies, in which a normal-appearing developmental myelination is followed by progressive demyelination and inflammation.

## Material and methods

### Human tissue and histology

Duplication of the *PLP1* locus was confirmed by quantitative reverse transcription-PCR as described previously (Inoue et *al*., 1999; Hobson *et al.*, 2000). PMD brains were removed at autopsy by J.G. (Sima *et al.*, 2009) and the cerebral and cerebellar hemispheres separated. One hemisphere from each region was fixed in 10% buffered formalin for histological analysis and the other was sliced in ∼1-cm thick sections and immediately snap frozen on dry ice and stored at −70°C. Several 1-cm axial sections of the spinal cord of Patient 1 were removed from different levels and frozen; the remainder were fixed in 10% buffered formalin. For the analysis described in this report, samples of the cerebral cortex, optic nerve, cerebellum and medulla from both brothers, and the cervical and lumbar cord from Patient 1 were used. Tissue was paraffin wax-embedded or cryoprotected in 20% sucrose and snap frozen. Selected regions were further fixed in glutaraldehyde, osmicated and processed for resin embedding. Paraffin sections were stained with haematoxylin and eosin or Holmes’ silver impregnation or immunostained by the PAP technique with rabbit anti-PLP1/DM20 (antibody to C-terminal, recognizing both isoforms), rabbit anti-PLP1 isoform (antibody to the PLP1-specific region), rat anti-myelin basic protein (MBP; Serotec MCA4095) and rabbit-glial fibrillary acidic protein (GFAP; Dako Z0334). Phosphorylated and non-phosphorylated epitopes on the neurofilament-H protein were immunostained with SMI-31 and SMI-32 (Covance mouse SMI31-R and SMI32-R) antibodies, respectively. Indirect immunofluorescence was also used to stain paraffin and cryosections. Control human material was obtained at autopsy. Respective CNS regions were fixed in formalin and embedded in paraffin. Paraffin sections were stained with haematoxylin and eosin and immunostained using the DAB technique for anti-myelin basic protein (MBP; Cell Marque) or with immunofluorescence with antibodies against phosphorylated neurofilament-H (Covance, SMI 31R) and MBP, respectively.

### Myelin isolation and western blotting

Frozen tissue obtained at autopsy, as described above, or fresh human control brain biopsy material that had been immediately frozen after isolation, was stored at −70°C. For myelin purification, tissue samples from the frontal lobes containing either a mixture of grey and white matter or white matter only were isolated while the tissue was still frozen, powdered with mortar and pestle in liquid nitrogen and 200 mg of material processed (Norton and Poduslo, 1973). In brief, tissue was homogenized using a Polytron blender in 0.85 M sucrose, 10 mM HEPES pH 7.4, 2 mM DTT and 1 mM TLCK. Sucrose (0.25 M) was gently layered on top of the homogenate, which was then centrifuged at 70 000*g* for 90 min at 4°C. The myelin interface was removed and hypotonically lysed in chilled dH_2_O and pelleted at 23 000*g* for 30 min at 4 C. Following an additional two rounds of hypotonic lysis, the myelin pellet was resuspended in 10 mM HEPES pH 7.4 containing 1 mM PMSF, 1 mM benzamidine, 10 μg/ml aprotinin and 10 μg/ml leupeptin. Myelin fractions were stored at −70°C. The protein concentration was determined using a Bio-Rad method using bovine serum albumin as a standard (Bio-Rad). Western blotting for PLP1/DM20, myelin-associated glycoprotein (MAG), MBP, and 2′,3′-cyclic-nucleotide 3′-phosphodiesterase (CNP) was performed using the enhanced chemiluminescence (ECL) method. The sensitivity was confirmed by loading different amounts of selected samples.

For control purposes, we used brain with no neurological disease, from a 70-year-old female who died of congestive heart failure and tissue (stored at −70°C) from a cerebral biopsy of a 2-year-old female undergoing surgery for removal of an epileptic focus that pathologically showed cortical gliosis. Normal-appearing white matter adjacent to the epileptic focus was immediately snap frozen for subsequent myelin preparation, as described above.

### Pelizaeus-Merzbacher disease mouse model

The *Plp1*-overexpressing line #72 (Readhead *et al.*, 1994) on a C57BL/6N background, *Thy-1-*CFP (Feng *et al.*, 2000), β-actin*-GFP* (Ikawa *et al.*, 1995) and *PLP1-LacZ* (Wight *et al.*, 1993) mice were used. The *Plp1*-tg #72 line harbours a cassette with three extra copies of the *Plp1* gene (Readhead *et al.*, 1994). Hemizygous mice were bred to generate homozygous transgene positive (*Plp1*-tg) and wild-type offspring. PCR was used to confirm the presence of the transgene and quantitative real-time PCR (outsourced to Embark Scientific) was used to ascertain gene copy number, to distinguish homozygous from hemizygous mice. For axon morphology, *Thy-1-CFP1* mice (B6.Cg-Tg Thy1-CFP 23Jrs/J) were crossed with *Plp1*-tg mice for at least six generations then homozygous *Plp1*-tg: hemizygous *Thy-1-CFP* offspring were used. Transplant recipients were male and female postnatal Day 1 (P1) or male P100 offspring of homozygous *Plp1*-tg parents.

Mice were bred in designated animal facilities, Biological Services, University of Glasgow (UoG). All experiments were conducted according to guidelines in the Animal’s Scientific Procedures Act 1986 under a project license (PPL 01/0507) from the UK Home Office, and approved by the local Ethics and Welfare review panel of the Faculty of Veterinary Medicine, UoG. Further details on housing are available in the online Supplementary material.

### Collection and preparation of animal tissue

Mice were rapidly perfusion fixed at ages P40, P60, P90 or P120 with 0.9% saline followed by 4% paraformaldehyde (PFA) for immunohistochemistry or 4% PFA/5% glutaraldehyde in 0.08 M cacodylate buffer (pH 7.2), 0.05% calcium chloride (‘strong’ fixative) for electron microscopy, immersed for >1 h in fixative, then dissected.

For immunohistochemistry, whole brain was cryoprotected in 20% sucrose/PBS, immersed in O.C.T. compound (Sakura Finetec) then snap frozen in liquid nitrogen-chilled isopentane and sectioned on an OTF cryostat (Bright Instruments) at 10 µm. For electron microscopy, brains were cut into ∼10 mm^3^ pieces, osmicated in 1% osmium tetroxide, dehydrated through a series of increasing concentrations of ethanol, cleared in propylene oxide, and embedded in Araldite^®^.

For light microscopy of resin-embedded tissue, sections were cut at 1 μm and stained with methylene blue (VWR)/azur II (VWR), then mounted on glass slides in DPX (VWR). For electron microscopy, 60 nm sections were stained with uranyl acetate (AGAR Scientific, R1260) and lead citrate (VWR, 290384R). Imaging was performed in the region where the fimbria merges with the corpus callosum, rostrodorsal to the hippocampus, using a JEOL 100CX electron microscope. After neurosphere transplantation, GFP or LacZ-positive tissue was post-fixed by immersion in ‘strong’ fixative for 1 week.

Antibodies and protocols for staining mouse tissue are provided in the Supplementary material.

### Neurospheres

Neurospheres were generated as described previously (Edgar *et al.*, 2004) from newborn homozygous *PLP-LacZ* or hemizygous β-actin-*GFP* mice. On the day of transplantation, *LacZ* and *GFP* expressing neurospheres were mixed together in ∼50:50 ratio, triturated lightly through a flame-polished glass pipette and resuspended following centrifugation in 20–30 µl ice-cold L15, corresponding to at least 50 000 cells/µl.

### Transplantation into the adult corpus callosum

Anaesthesia was induced in P100 mice with 5% isoflurane and maintained with 2% isoflurane, both in 30% O_2_/70% NO_2_. Deeply anaesthetized animals were fixed in a rodent stereotactic frame. Right-sided craniectomies were performed, and a glass capillary connected to a microinfusion pump (CellTram Oil manual piston pump, Eppendorf) was placed according to stereotactic coordinates (Paxinos and Franklin, 2001) −0.58 mm or 1.34 mm caudal to bregma, and 0.5 mm lateral to the midline suture and 1.5 mm ventrally to the brain surface. A 1 μl neurosphere suspension was injected at 0.1 μl/min. Pre- and post-surgical analgesia was provided.

### Transplantation in neonates

Newborn pups (P1) were anaesthetized with 5% isoflurane in O_2_ and transferred to a 37°C heating pad. The head was fixed gently between the operator’s fingers and four injections were made using a tapered 30-G needle attached to a 5 μl Hamilton syringe (Hamilton 65RN) to a depth of 1.5 mm, immediately right and left of bregma point 0, and immediately right and left of lambda. A 0.5 μl neurosphere suspension was administered slowly at each site.

### Administration of bromodeoxyuridine

A single intraperitoneal injection of 50 µg/g body weight 5-bromo-2-deoxyuridine (BrdU; Sigma-Aldrich, Cat. B5002) in physiological saline was administered 1 h prior to perfusion fixation in 4% PFA.

### Cell quantification

Cell densities were measured on micrographs on which a rectangular area of interest of known area was placed. All cells with a nucleus within or whose nucleus crossed south and west borders were counted.

Quantification of cells labelled using immunofluorescence was performed in the corpus callosum on 10-µm thick transverse cryosections. Six or more sections, at 100-µm intervals, were stained with each antibody. One image per section was taken at ×40 magnification; six or more images per antibody were quantified as described previously (Edgar *et al.*, 2010).

Quantification of cells in resin sections was performed in the corpus callosum on 375–500-nm thick sections stained with methylene blue/azur II, imaged using a 100× oil objective. Cells were identified as activated microglia/macrophages on the basis of having a dense, dark cytoplasm, which was obvious at the level of the light microscope, and a characteristic ‘angular’ shape.

Electron microscopy to identify cell types was done on a JEOL 100CX microscope with digital camera. Images were captured at ×600, ×1000 and ×1500 magnification to allow characteristic features (rough endoplasmic reticulum, Golgi apparatus, mitochondria, microtubules, lysosomes, glial filaments) to be observed.

### Morphometry from electron micrographs

Eight electron micrographs per animal were captured at random from the mid corpus callosum at ×8000 using a film camera (JEOL 100CX). Negatives were scanned (Epson Perfection 3200 Photo) and images evaluated in ImagePro 6.0. Total axon densities were counted within, and on north and west borders of 45-µm^2^ areas of interest. For percentage myelinated axons, axons were quantified if they occurred on four overlaid bisecting lines and ascribed as myelinated or non-myelinated.

### Oligodendrocyte precursor cell culture and assessment of process length

For each adult oligodendrocyte precursor cell (aOPC) co-culture, two adult wild-type GFP-expressing and two adult *Plp1*-tg corpus callosa were used. *Plp1*-tg and GFP-expressing wild-type OPCs were mixed in equal numbers and plated at a concentration of 10^5^ cells/13-mm diameter glass coverslip coated with poly-l-lysine. OPCs were obtained from whole brains of P5 mice, as above, but cells were plated at 3–4 × 10^4^ cells/coverslip. Adult and neonatal OPCs were obtained using identical dissociation and centrifugation protocols. See Supplementary material for further details.

To evaluate total process length per cell, six semi-randomly chosen fields per coverslip were photographed using phase and fluorescence microscopy (Olympus IX-70, ×20 objective). Semiautomatic tracing of OPC processes was carried out in ImageJ using the NeuronJ plug-in on phase microscopy images.

### Statistical analysis and graphical presentation of the data

An unpaired Student’s *t*-test was used to compare cell densities, axonal densities and percentage myelinated axons. Statistical analysis and the graphical presentation of data were performed using GraphPad Prism 5^®^. For OPC process length comparison, a two-tailed Mann-Whitney test was used as the data were not normally distributed. This analysis was done using GraphPad Prism 8^®^. *P*-values are indicated as *P <* *0.05, **P <* *0.01, ***P <* *0.001 and ****P <* *0.0001.

### Data availability

The data that support the findings of this study are available from the corresponding author, upon reasonable request.

## Results

### Clinical and MRI assessment is compatible with CNS hypomyelination

The patients described here are members of a family previously described (Watanabe *et al.*, 1973; Wilkus and Farrell, 1976), and are in the sibship of three brothers, two of whom were affected (Generation VI). The duplication is 509 297 bp in size (Woodward *et al.*, 2005; Patient P116). Quantitative RT-PCR analysis demonstrated an ∼1.25-fold higher level of *PLP1* gene expression than in a patient with multiple sclerosis (Supplementary Table 1).

### Patient 1

After an uncomplicated full-term pregnancy and delivery, Patient 1 was noted to have nystagmus within the first 2 weeks after birth. By 6 months of age it was apparent his gross motor development was delayed. He was hypotonic during infancy and required head support. He was delayed for rolling over and was not able to sit without support until ∼30 months old. He was never able to walk. Spasticity was noted beginning at about age 15 and progressed, necessitating tendon releases in the hip adductors in the fourth and fifth decades. The patient could perform simple tasks such as finger feeding, but movements were ataxic. By ∼30 years of age he required full assistance for feeding. Speech developed at a normal age and until about age 17 he was able to verbally communicate effectively. After that age, speech became progressively more dysarthric and he was unable to speak after about age 30. Cognition was impaired and the patient was educated in special education settings until transferred to an institution for developmentally impaired children when he was 12 years old. He remained incontinent throughout his life. He enjoyed watching sports and recognized names of the local sporting teams. The patient was examined at age 51 (by J.G.). At that time the patient was alert and could recognize family members and caretakers. He was able to perform simple commands, but speech was absent. Most communication was through hand signals and facial expression. He was able to communicate using an electronic touch-sensitive communication board. Head titubation and compensatory nystagmus were present when his head was not supported while sitting, but both ceased when his head was supported. Upper extremity strength was 3/5 on the MRC scale, with spasticity and contractures at the biceps and wrist flexors. The patient required a thoracic brace and headrest to maintain upright posture while sitting in his wheelchair. The legs were very atrophic, but no fasciculations were noted. There was no voluntary leg movement. Reflexes were abnormally brisk and Babinski signs and triple flexion were elicited on plantar stimulation. Arm movements were slow and ataxic. The patient required a feeding tube for the last year of life and died of aspiration pneumonia at age 54.

### Patient 2

**Figure 1:**
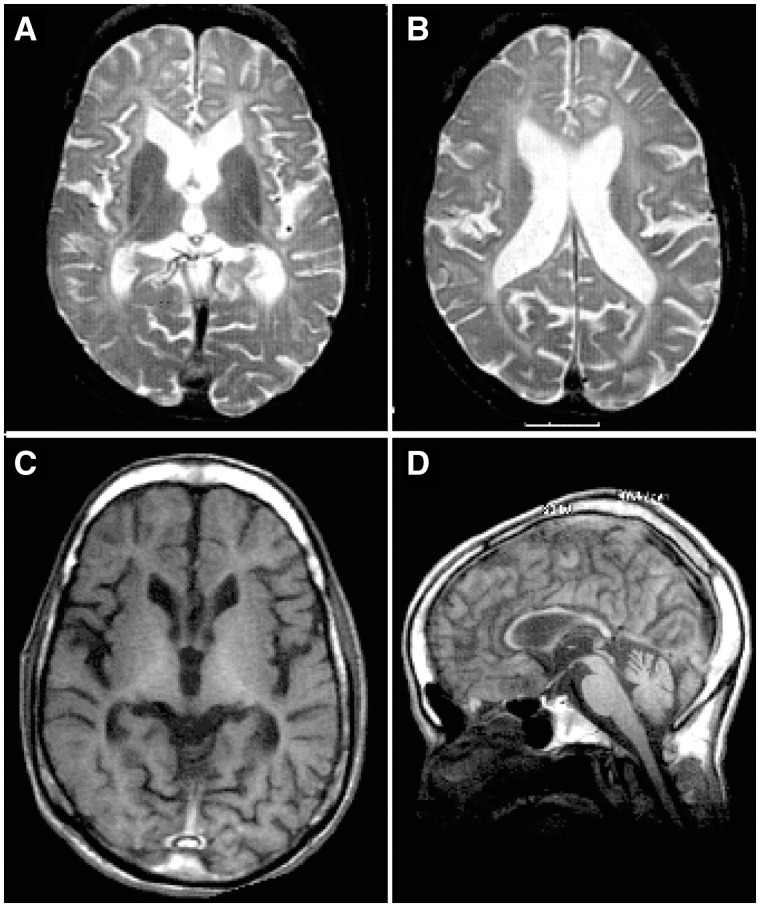
**MRI scan of Patient 2 at age 41 illustrates reduced white matter.** (**A** and **B**) T_2_-weighted axial sections showing the diffusely increased signal in the cerebral white matter; however, there is a thin, normal signal in the corpus callosum and the internal capsule. (**C** and **D**) T_1_-weighted images showing the reduced amount of white matter: note the thinness of the corpus callosum and of the fingers of white matter extending into cortical gyri in **C**. Thinning of the corpus callosum and of the cervical spinal cord can be observed in **D**. Reference T_1_ and T_2_ MRI images of healthy individuals can be found in publicly available MRI brain atlases, such as the Whole Brain Atlas by the Harvard Medical School http://www.med.harvard.edu/AANLIB/cases/caseNA/pb9.htm or the IMAIOS webservice https://www.imaios.com/en.

Patient 2 had a similar clinical course except that his mother did not note nystagmus at any age. Clinical examination of Patient 2 at age 45 was essentially the same as that of his brother. He also required a feeding tube for the last year of life and he expired at age 47 of aspiration pneumonia. An MRI scan at age 41 (Fig. 1) showed diffuse white matter signal hyperintensity on T_2_-weighted images with enlarged ventricles and reduced white matter volume (Fig. 1A and B). The T_1_ signal was also diffusely reduced (Fig. 1C), and the corpus callosum markedly thinned (Fig. 1D).

**Figure 2:**
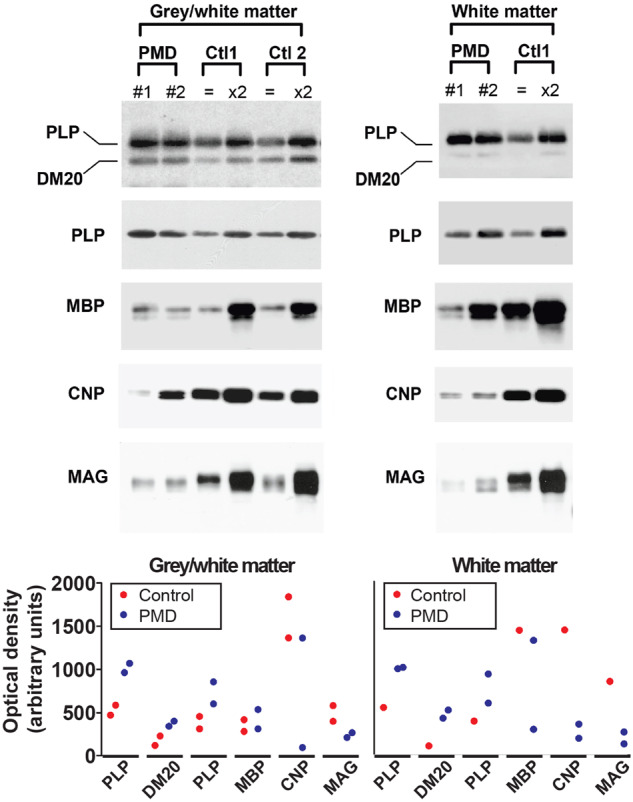
**Western blot of myelin extracts from PMD and control human brain show altered levels of myelin proteins.** Ctl1 is stored tissue from 70-year-old brain and Ctl2 is fresh biopsy material from 2-year-old brain white matter. Myelin was extracted from tissue that was composed of a mixture of grey and white matter or tissue that was composed almost entirely of white matter. For PLP and MBP analysis, 0.25 μg of the PMD myelin was loaded; in the control patients 0.25 μg (equal symbol) and 0.5 μg (×2) of the samples were loaded onto the gel. For CNP and MAG analysis, 1.5 μg of the PMD myelin and 1.5 μg (equal symbol) and 3 μg (×2) of the control samples were loaded onto the gel. Graphs provide semiquantitative data reflecting band intensities. The *y*-axis values are the same in the two graphs. Blots were cropped for display purposes. Full-length blots are available in the Supplementary material.

To determine how *PLP1* duplication affected levels of PLP/DM20 and other major myelin proteins, we used western blotting. There was no evidence of protein degradation in the patients’ samples, assessed against fresh biopsy material. Myelin preparations from mixed grey and white matter or almost entirely white matter were assessed. The levels of the *PLP1* and DM20 isoforms were approximately twice the amount observed in the controls. MBP levels were comparable between the PMD and controls whereas the levels of CNP and MAG, two proteins of non-compact myelin, were markedly reduced in the PMD samples (Fig. 2).

In summary, the clinical and MRI evaluations were consistent with classical PMD, and demonstrated a slowly progressive deterioration of neurological function. Biochemical analysis confirmed increased levels of PLP/DM20 in PMD brain, as anticipated from the molecular diagnosis.

### Pathology involves dysmyelination, demyelination and axon degeneration

**Figure 3:**
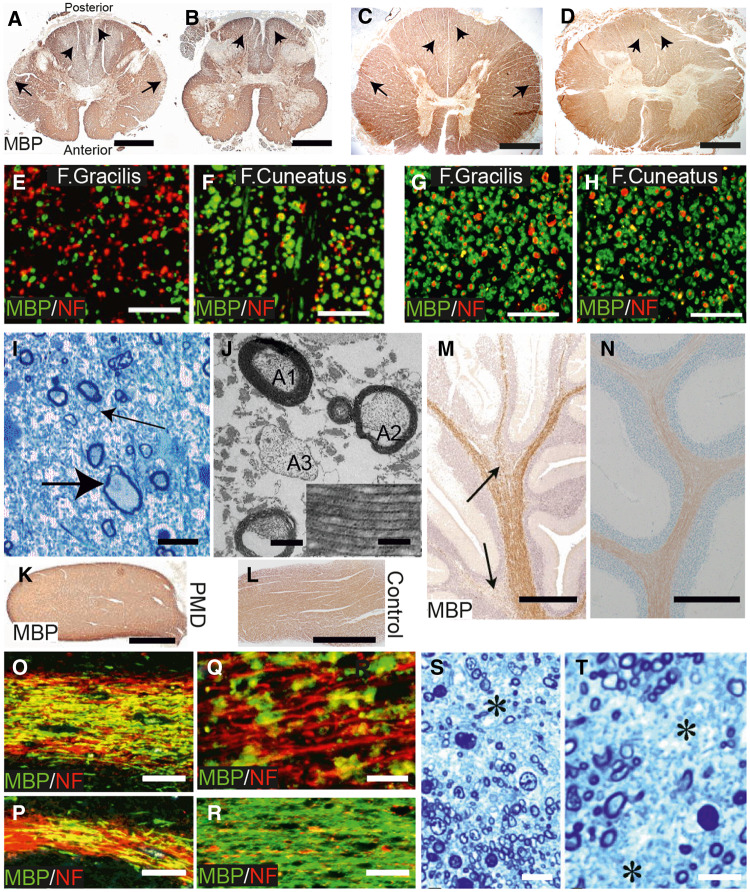
**Evidence for dysmyelination, demyelination and axon degeneration in PMD.** (**A** and **B**) Cervical and lumbar cord (Patient 1) stained for MBP. While the majority of white matter is stained strongly, symmetrical areas of pallor are present in the central posterior columns (short arrows) and the periphery of the lateral columns (arrows) in **A** and in the posterior columns (short arrows) in **B**. Scale bars = 2 mm. (**C** and **D**) Sections from control for comparison. Scale bars = 2 mm. (**E** and **F**) Regions of the fasciculus gracilis (F. gracilis) and peripheral fasciculus cuneatus (F. cuneatus) double immunostained for MBP (green) and phosphorylated heavy chain neurofilament (NF, red). (**G** and **H**) Sections from control for comparison. Scale bars = 50 µm. There are a reduced number of fibres in the F. gracilis and many axons have no myelin sheath. In the F. cuneatus the majority of axons have a myelin sheath. (**I**) Resin section from periphery of lateral columns. There is a marked reduction in myelinated nerve fibres and replacement by astrocytic processes. A swollen axon (large arrow) and axons with thin myelin sheaths (small arrow) are present. Scale bar = 10 μm. (**J**) Electron micrograph from one of the areas of MBP pallor. Myelinated fibres are reduced and astrocyte processes are increased. One axon (A1) has a myelin sheath of normal thickness; another (A2) has a thin sheath; a third (A3) has no myelin sheath. Scale bar = 1 μm. *Inset*: Regular structure of major dense and intraperiod lines. Scale bar = 0.05 μm. (**K** and **L**) PMD and control optic nerves, immunostained for MBP. Strong, generalized staining is present in both. Scale bars = 1 mm. (**M**) Area from PMD cerebellum immunostained for MBP. The white matter is relatively well stained in the central part of the folium, tending to decrease in the branches. Two regions of more reduced MBP staining are present (arrows). Scale bar = 1 mm. (**N**) Area from cerebellum from control for comparison. Scale bar = 1 mm. (**O** and **P**) Cerebellar white matter from folia stained by double immunofluorescence for MBP (green) and NF (red). In **O**, only a minority of axons are lacking myelin whereas in **P**, an area of naked axons is present on the left with an island of myelin in the centre. Scale bars = 50 μm. (**Q**) Higher magnification image. Few axons have an intact myelin sheath and the myelin is present as globules, suggesting demyelination. Scale bar = 20 μm. (**R**) Cerebellar white matter from control for comparison with **O** and **P**. Scale bar = 50 μm. (**S** and **T**) Resin sections of the PMD corpus callosum. There is a considerable loss of myelinated axons (asterisks) with replacement by astrocytic processes. Scale bars = 10 μm.

Examination of the brains at autopsy revealed generalized reduction of cerebral white matter with relative sparing of U-fibres. The cortical ribbon was of appropriate thickness. Cerebellar white matter was globally reduced. There was marked atrophy of the cerebral peduncles and corticospinal tracts. Pigmentation of the substantia nigra was reduced. Neuronal loss and gliosis were noted in several regions including substantia nigra, amygdala, anterior striatum and in entorhinal, anterior cingulate, insular and parietal cortices and the thalamus, in particular the pulvinar and dorsal medial nucleus. The spinal cord of Patient 1 was severely atrophied being ∼6 mm in diameter at the cervical and lumbar levels (Fig. 3), compared to an average of 7.7 and 8.0 mm, respectively, reported previously (Elliott, 1945). A more detailed analysis of the neuronal changes is presented elsewhere (Sima *et al.*, 2009).

Based on the above, we selected specific regions for a more detailed analysis of myelin and axonal changes. Histological findings in the material common to both brothers were similar. Paraffin sections of spinal cord generally stained strongly for both *PLP1*/DM20 and MBP although areas of pallor were present in the centre of the posterior columns and the periphery of the lateral columns (Fig. 3A and B). Control spinal cord white matter was evenly stained (Fig. 3C and D). Outside of the zones of pallor, the density of nerve fibres appeared well preserved and the vast majority of axons had myelin sheaths of appropriate thickness. The difference between regions of strong and reduced myelin protein immunostaining was exemplified in the posterior columns contrasting the fasciculus gracilis, where there were large numbers of demyelinated axons (Fig. 3E), with the peripheral area of the fasciculus cuneatus, where the vast majority of axons were well myelinated (Fig. 3F). Control material is shown for comparison (Fig. 3G and H). Within the areas of decreased immunostaining there was a marked loss of fibres and a corresponding increase in astrocytic processes; corpora amylacea were also common (Fig. 3I). Fibres with thin myelin sheaths or naked axons were observed (Fig. 3J). The myelin present was compacted and demonstrated major dense and intraperiod lines (Fig. 3J, inset). Swollen axons were seen infrequently (Fig. 3I). Myelin debris was infrequent and macrophages were not a feature in these regions of spinal cord. Paraffin sections of optic nerve immunostained strongly for *PLP1*/DM20 (not shown) and MBP (Fig. 3K), similar to that of a control (Fig. 3L). Immunostaining for MBP and *PLP1*/DM20 was present throughout the medulla, cerebellum and cerebral cortex/subcortical white matter; however, there was considerable variation within regions. For example, in the cerebellar white matter the extent of staining tended to decrease into the folia (Fig. 3M), which was not the case in the control, as expected (Fig. 3N). Double immunostaining for MBP and SMI-31 (phosphorylated heavy chain neurofilament) suggested that much of this patchy distribution was due to absence of myelin from axons (Fig. 3O and P). Additionally, some of the MBP-stained myelin was not associated with axons and appeared to be present as globules, suggesting a degree of demyelination (Fig. 3Q). In contrast, control cerebellum MBP staining was largely homogenous (Fig. 3R). In PMD corpus callosum there was clear evidence for axonal loss with replacement by glial processes (Fig. 3S and T).

### Developmental myelination proceeds almost normally in a mouse model of *PLP1* duplication mutation

**Figure 4:**
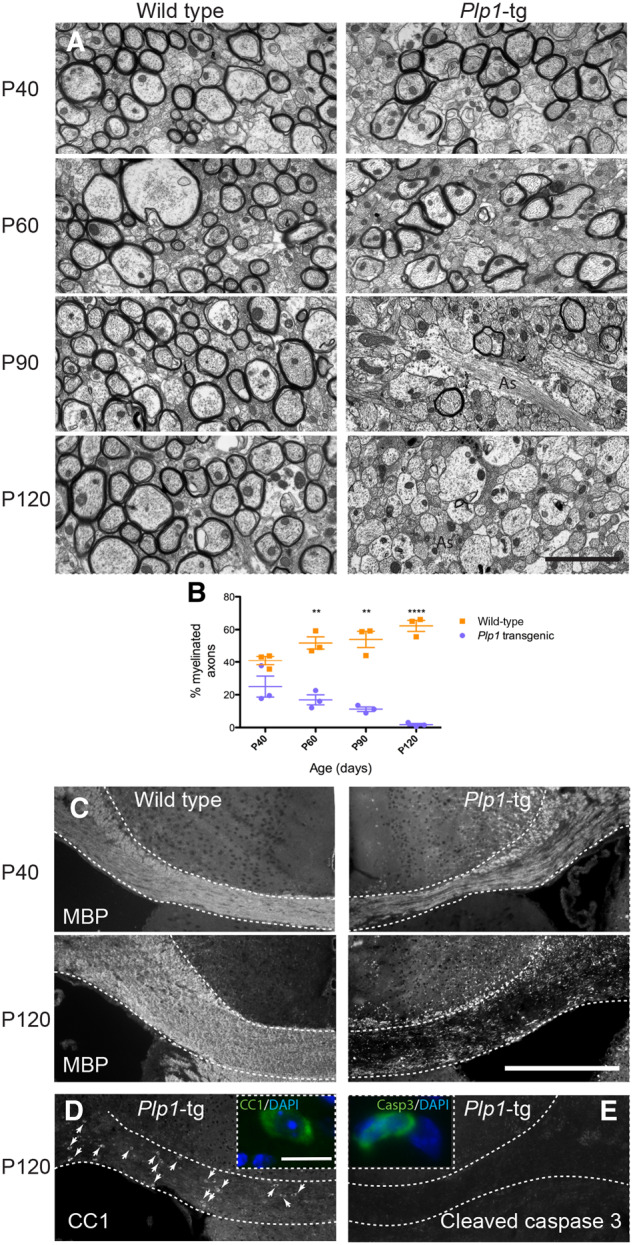
**Progressive demyelination in *Plp1*-tg mouse brain.** (**A**) Electron micrographs of the corpus callosum in wild-type and *Plp1*-tg mice between P40 and P120. In *Plp1*-tg mice, at P40 and to a lesser extent at P60, many axons are surrounded by compact myelin, which appears ultra-structurally normal. The proportion of myelinated axons is markedly diminished by P90 and virtually all axons are devoid of myelin sheaths by P120. Astrocyte processes (as) are particularly evident in *Plp1*-tg corpus callosum at P90 and P120. (**B**) Graph demonstrating that the proportion of myelinated axons in *Plp1*-tg mice is similar to wild-type at P40, but diminishes over time. Bars represent mean ± standard error of the mean (SEM). (**C**) Immunohistochemistry using antibody to MBP at P120 demonstrates the extent of myelin loss across the corpus callosum in *Plp1*-tg mice. (**D**) Antibody CC1-positive mature oligodendrocytes are still present in *Plp1*-tg corpus callosum at P120, suggesting myelin loss is not, or only partially due to the death of oligodendrocytes. (**E**) Accordingly, most sections of the corpus callosum contained no cleaved caspase 3-positive cells. The positive cell shown in the *inset* is one of the few cells observed. Scale bars = 2 μm in **A**; 400 μm in **C**; 10 μm in *inset* **D.**

To assess if direct cell transplantation into the CNS might benefit patients with classical forms of PMD, we turned to a *Plp1*-tg mouse model. The pathology and phenotype have been overviewed previously (Readhead *et al.*, 1994; Anderson *et al.*, 1999) but not characterized systematically. Mice appeared almost indistinguishable from wild-type littermates until around P90, after which they displayed a mild ataxia and exhibited seizure activity. During the next month of life, the generalized ataxia became progressively more pronounced, a mild whole-body intention tremor developed, and seizure activity increased in frequency. As described previously, correlative histology and diffusion tensor imaging at P120 revealed profound hypomyelination throughout the brain, including grey and white matter (Ruest *et al.*, 2011), although the spinal cord remained well myelinated at this age (data not shown). To define the evolution of the pathology associated with these progressive neurological signs we used electron microscopy to examine myelinated axons in the mid corpus callosum between P40 and P120 (Fig. 4A). At P40, myelin appeared normal at the ultrastructural level and the percentage of axons with a myelin sheath was similar to wild-type. However, although axon density did not change compared to control (Supplementary Fig. 1), the percentage of myelinated axons decreased progressively over time until P120 when myelin sheaths were virtually absent (Fig. 4A and B). This progressive demyelination was reflected, at the level of the light microscope, in a marked reduction in MBP immunostaining between P40 and P120 (Fig. 4C). Despite the loss of myelin, mature oligodendrocytes (identified with antibody CC1) were present in the corpus callosum of *Plp1*-tg mice at all ages examined, including P120 (Fig. 4D, arrows) although the number of CC1-positive cells appeared slightly diminished compared to wild-type at P90 and P120 (Supplementary Fig. 1). These data suggest demyelination is not, or only partly, due to oligodendrocyte cell death. Correspondingly, caspase 3-positive apoptotic cells were observed only occasionally in *Plp1*-tg mouse corpus callosum (Fig. 4E).

These changes in myelination were associated with significantly increased amounts of GFAP and of CD45-positive microglia/macrophages from P60 onwards (data not shown), and the appearance of sialoadhesin (CD169)-positive microglia/macrophages and a small number of CD3-positive T cells from P90 (Supplementary Fig. 2). Furthermore, focal axon swellings were observed at P120, as visualized in *Plp1*-tg::*Thy1-YFP* mice (Supplementary Fig. 2). At this age, *Plp1*-tg mice had to be euthanized under the conditions of the UK Home Office project licence.

In summary, normal-appearing developmental myelination is followed by progressive demyelination, which is accompanied by astrocytosis, neuroinflammation and axonal changes. Notably, progressive demyelination has been reported in other proteolipid-overexpressing models (Mastronardi *et al.*, 1993; Ma *et al.*, 2006).

### *Plp1*-tg oligodendrocyte precursor cells are present throughout the disease course in mice

**Figure 5:**
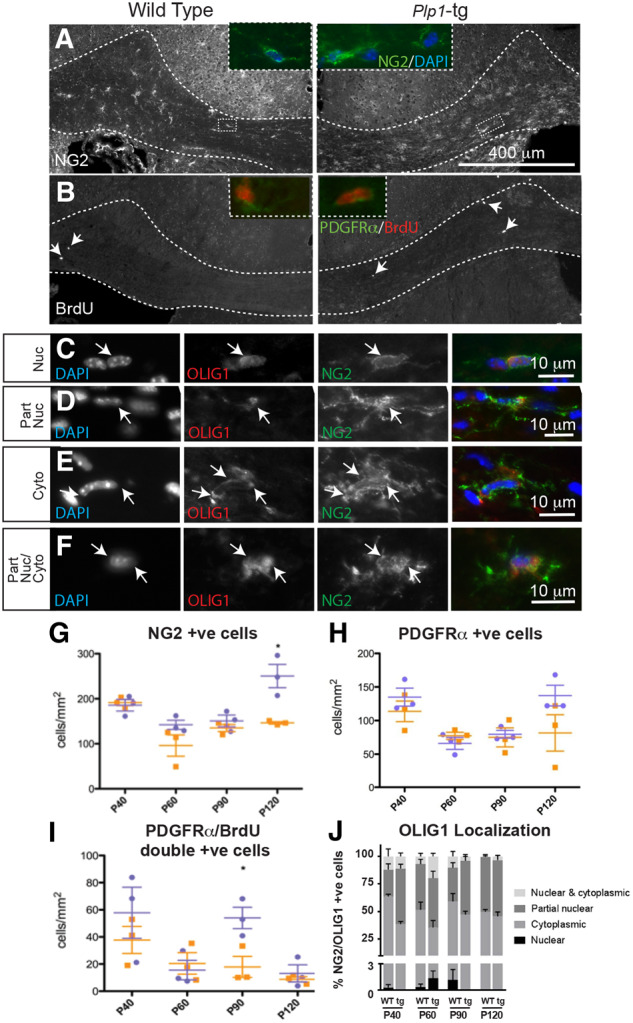
**Plp-tg mouse OPCs in adult brain mount a limited response to demyelination.** (**A** and **G**) NG2 or (**B** *inset*, and **H**) PDGFRα-positive OPCs were present in both wild-type and *Plp1*-tg mouse corpus callosum at all ages examined, with NG2-positive cells being at slightly but significantly higher density in the P120 *Plp1*-tg animal compared to the age-matched control (**G** and **H**). Wild-type is shown in orange, *Plp1*-tg in blue. Bars represent mean ± SEM. (**B** and **I**) In both *Plp1*-tg and control animals, a small number of BrdU-positive cells were present in the corpus callosum at 1-h post-BrdU administration. (**I**) At P90, the number of PDGFRα-positive cells double labelled with anti-BrdU was significantly higher in the *Plp1*-tg animals compared to control (wild-type in orange, *Plp1*-tg in blue). Bars represent mean ± SEM. (**C**–**F** and **J**) The basic helix-loop-helix transcription factor OLIG1 translocates to the nucleus during OPC differentiation and the subcellular localization of the transcription factor can be used to assess the status of the OPCs. In *Plp1*-tg corpus callosum, a small proportion of NG2-positive cells had a nuclear localization of OLIG1 (**C** and **J**). Most NG2-positive cells had a partially nuclear (**D** and **J**), cytoplasmic (**E** and **J**), or partially nuclear and partially cytoplasmic (**F** and **J**) localization. Images were adjusted manually for brightness and contrast in Adobe Photoshop for ease of visualization.

These results demonstrate that *Plp1*-tg oligodendrocytes are myelination-competent. Moreover, stained sections from brains at ages P40, P60, P90 and P120, using antibodies against NG2 (Fig. 5A) or PDGFRα (Fig. 5B), revealed a density of OPCs similar to that in wild-type animals, and possibly even increased compared to wild-type at P120 (Fig. 5G and H). The latter may be due to an increase in proliferating PDGFRα-positive OPCs at P90 (Fig. 5B andI). Taken together, normal-appearing early myelination could prevent a successful transplantation therapy with exogenous OPCs, due to the competitive advantage of the endogenous OPCs and oligodendrocytes.

However, in light of the progressive loss of myelin (Fig. 4), these data raise the possibility that unlike OPCs in neonatal *Plp1*-tg mice (nOPC), mutant aOPCs may lose the competence to synthesize myelin. To address this, we examined the intracellular localization of the basic helix-loop-helix transcription factor, OLIG1, which translocates from the oligodendroglial cytoplasm into the nucleus during remyelination. Indeed, failure of this intracellular redistribution is associated with remyelination failure in multiple sclerosis (Arnett *et al.*, 2004). As expected, in *Plp1*-tg mice at age P3, we found that the endogenous nOPCs exhibit an intranuclear localization of OLIG1 (data not shown), whereas in adult *Plp1*-tg mice, <3% of NG2-positive OPCs had a pure nuclear localization at any age (Fig. 5C and J). In the adult brains, similar to wild-type controls, OLIG1 localization in NG2-positive OPCs was partially nuclear (Fig. 5D and J), purely cytoplasmic (Fig. 5E and J); or partially nuclear and partially cytoplasmic (Fig. 5F and J).

Collectively, these data are compatible with the absence of effective myelin repair of the progressively demyelinating adult *Plp1*-tg CNS, but do not allow us to distinguish aOPC-intrinsic failure to remyelinate from an inhibitory CNS environment for remyelination. These factors clearly affect the likelihood of success of any cell transplantation therapy.

### Direct transplantation of neural precursors into *Plp1*-tg brain results in formation of compact myelin

**Figure 6:**
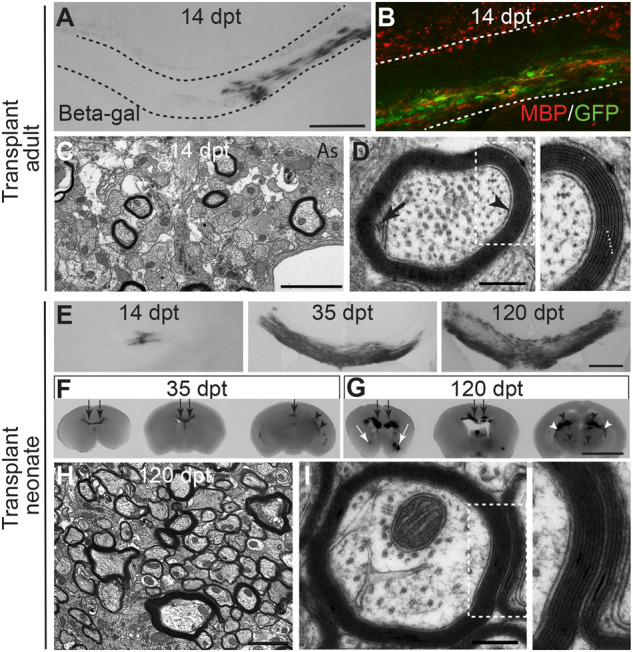
**Neural precursors form compact myelin sheaths after transplantation into adult or neonatal *Plp1*-tg mice.** (**A**) Light microscopy image of beta galactosidase-positive cells in the corpus callosum (delineated) of a *Plp1*-tg transplant recipient, 14 days post transplantation of *PLP1-lacZ* and β-actin GFP-expressing neurospheres into the brain at P100. (**B**) Corpus callosum of another transplant recipient showing that transplanted GFP-positive neurospheres form MBP-positive myelin. The red staining above the upper line delineating the corpus callosum is from endogenous myelin debris. (**C** and **D**) Electron micrographs showing that the transplanted cells generate oligodendrocytes that wrap axons with normal-appearing myelin sheaths in which the inner tongue (black arrow), periaxonal space (arrowhead) major dense lines (asterisks in **D** *inset*) and intraperiod lines (dots in **D** *inset*) can be observed. Scale bars = 400 μm in **A**; 2 μm in **C**; and 250 nm in **D**. (**E**) Light microscopy image of beta galactosidase-positive cells in the corpus callosum of *Plp1*-tg transplant recipients at 14, 35 and 120 dpt of *PLP1-lacZ* expressing neurospheres into the brain at P1. (**F** and **G**) Slices of transplant recipient brains (1-mm thick, presented rostral to caudal) at 35 dpt (**F**) and 120 dpt (**G**), to illustrate how the transplanted cells distributed throughout the brain including the corpus callosum (black arrow), anterior commissure (white arrow), thalamus (black arrowhead) and fimbria of the hippocampus (white arrowhead). (**H** and **I**) Electron micrographs showing that the transplanted cells wrap axons with normal-appearing myelin sheaths in which the periaxonal space, major dense lines and intraperiod lines can be observed. Scale bars = 400 μm in **A** and **E**; 2 μm in **C** and **H**; 250 nm in **D** and **I**; and 5 mm in **F** and **G.**

To determine if the environment of the adult *Plp1*-tg mouse brain allows (re)myelination, we transplanted a combination of neurospheres expressing either *GFP* under the chicken β-actin promoter or the *lacZ* reporter gene under the *PLP1* promoter into the brains of adult (P100) *Plp1*-tg mice. Importantly, 14 days later, the transplanted cells appeared fully integrated (Fig. 6A and B) and had formed compact myelin around a small number of axons in the vicinity of the transplant site (Fig. 6C and D), providing proof-of-principle that the adult *Plp1*-tg brain remains myelination-competent. However, a therapeutically more plausible approach would be to transplant cells early in development. To test if the exogenous cells can survive and myelinate axons in the face of competition from the endogenous, myelination-competent *Plp1*-tg nOPCs, we next transplanted neurospheres to the brains of P1 *Plp1*-tg mouse pups. At each of 7, 14, 35 and 120 days post-transplant (dpt), two mice were sacrificed and 1-mm thick slices of brain were stained with X-gal and evaluated. No transplanted cells were visible at 7 dpt. However, at 14 dpt, a few isolated β-galactosidase positive cells with myelin sheath-like morphologies were observed (Fig. 6E) and at 35 (Fig. 6E and F) and particularly at 120 dpt (Fig. 6E and G) large patches of X-gal staining were observed throughout the brains including the corpus callosum, anterior commissure, thalamus, hypothalamus, optic chiasm and hippocampus. Electron micrographs of the corpus callosum of transplant recipients demonstrated that compact myelin had formed around numerous axons (Fig. 6H andI).

Unsurprisingly, the mice that received cell transplantation at P100 did not have altered clinical signs. However, those treated as neonates appeared normal at the point of euthanasia, including an absence of seizures in the two animals euthanized at P120. Under the conditions of our UK Home Office project licence we were unable to maintain the animals beyond that point.

### *Plp1*-tg adult but not newborn oligodendrocyte precursor cells fail to differentiate normally *in vitro*

**Figure 7:**
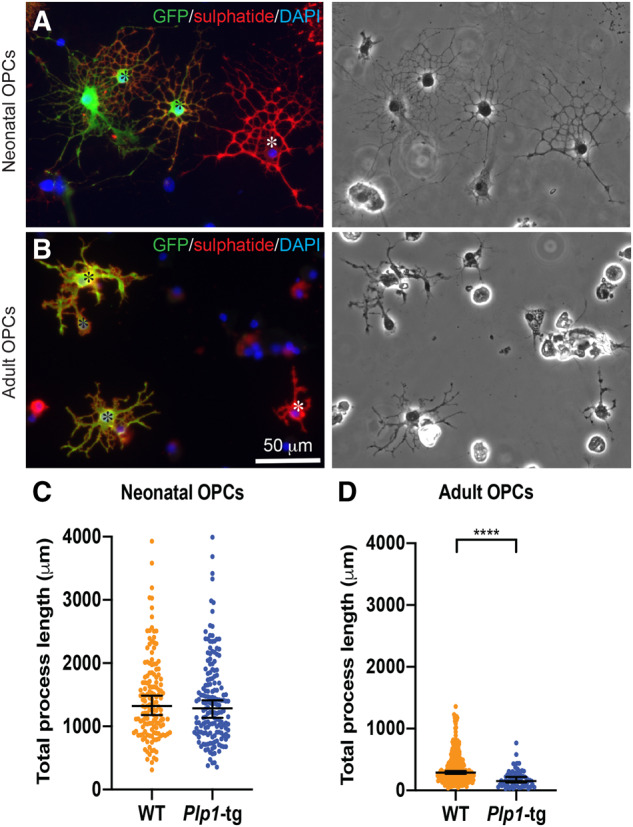
**Cell intrinsic factors prevent adult *Plp1*-tg OPCS from differentiating normally.** (**A** and **B**) GFP-positive wild-type (WT) (black asterisks) and GFP-negative *Plp1*-tg OPCS (white asterisks) were isolated from P5 forebrain (**A**) or adult corpus callosum (**B**) and co-cultured for 7 days. (**C** and **D**) Graphs of total process length per cell was measured from phase-contrast images of anti-sulphatide-positive cells. There was no difference in mean total process length of neonatal-derived *Plp1*-tg OPCs (**C**), which form normal-appearing myelin *in vivo*, compared to wild-type cells (Fig. 4). However, the mean total process length of *Plp1*-tg aOPCs was significantly less than that of wild-type aOPCs (*P *<* *0.0001; **D**). Each data point represents one cell, from two or three independent cultures, respectively. Each cell was considered independent for the purpose of the statistical analysis. Bars represent mean ± SEM. Note the different scales on the *y*-axes, demonstrating that even wild-type aOPCs fail to expand processes to the same extent as wild-type nOPCs. The images on the *left* were adjusted for brightness and contrast using ‘auto’ in Adobe Photoshop for ease of visualization.

Given that *Plp1*-tg nOPCs form almost normal amounts of myelin *in vivo*, we were surprised that cell transplantation into neonatal *Plp1*-tg mice was so successful. Because we had previously shown that oligodendrocyte survival and myelination in the *Plp1* mutants is influenced by ‘competing’ oligodendrocytes (Edgar *et al.*, 2002) we next compared the intrinsic differentiation potential of neonatal or adult *Plp1*-tg OPCs to their wild-type counterparts, in cell culture. We co-cultured *Plp1*-tg nOPCs (Fig. 7A) or *Plp1*-tg aOPCs (Fig. 7B) with age-matched GFP-expressing wild-type cells for up to 7 days then measured the extent of process extension. The mean total process length of anti-sulphatide (clone O4) positive *Plp1*-tg nOPCs was similar to controls (Fig. 7A and C), suggesting there is no competitive advantage for the wild-type nOPCs with respect to their ability to extend myelin-like sheets. However, the mean total process length of *Plp1*-tg aOPCs was significantly reduced compared to controls (Fig. 7B and D). These data suggest that intrinsic properties of *Plp1*-tg aOPCs account for or contribute to the failure of effective remyelination in *Plp1*-tg mice, and could provide a competitive advantage for exogenous wild-type OPCs to wrap axons, following transplantation in neonates or adults.

### Cell transplantation reduces neuroinflammation

**Figure 8:**
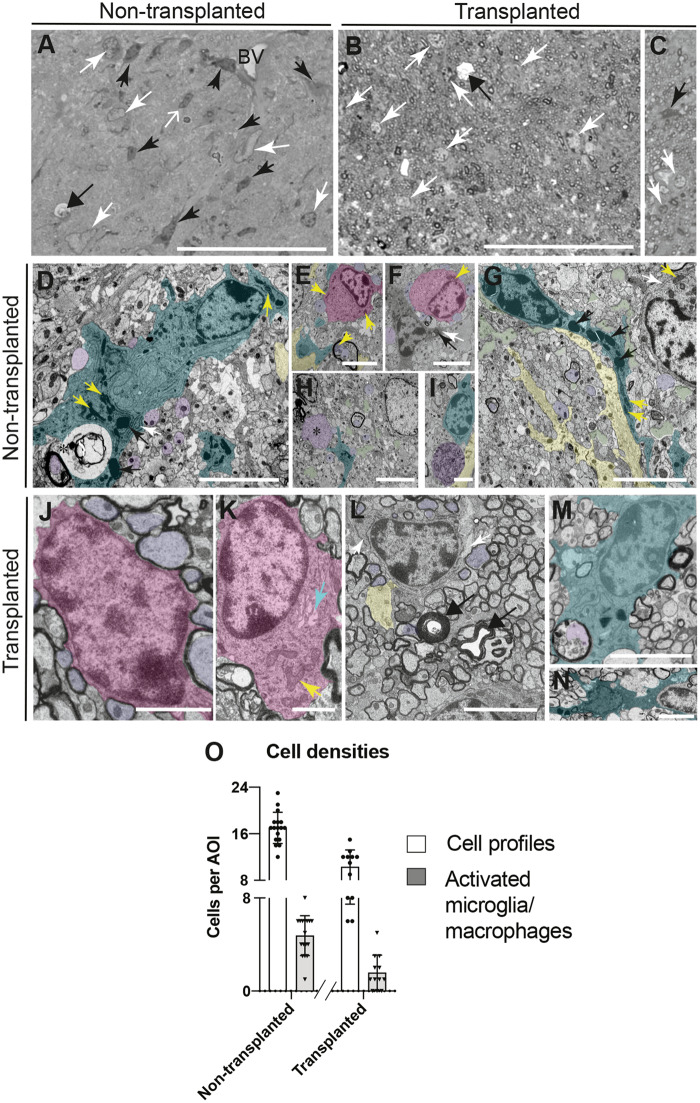
**Neuroinflammation is reduced after transplantation.** (**A**–**C**) Light microscopy of resin sections of corpus callosum of untreated (**A**) and transplant recipient (**B** and **C**) *Plp1*-tg mice at P120. Narrow black and white arrows indicate cell profiles, some of which (black arrows) can be identified as microglia/macrophages, due to having a dark nucleus and dark cytoplasm. Cells with a dense nucleus but without a dense cytoplasm (open white arrow in **A**) were not classified as microglia/macrophages. Occasionally, demyelinating axons (wide arrows in **A** and **B**) were observed. Scale bar = 50 μm. (**D**) Microglia/macrophage (overlaid in turquoise) containing dense lysosomes (black arrows), prominent mitochondria (yellow arrows) and rough endoplasmic reticulum (rER; white arrows) apparently engulfing degenerating myelin (asterisk). Normal-appearing axons are also present (purple); one with a myelin sheath (*lower left*). Scale bar = 5 μm. (**E**) An oligodendrocyte (pink) in apposition to non-myelinated axons (purple). In the *lower middle*, an axon (purple) containing large mitochondria (yellow arrow), is surrounded by degenerate myelin. Microglial/macrophage and astrocyte processes, turquoise and yellow respectively. Scale bar = 5 μm. (**F**) Oligodendrocyte (pink) in close apposition to non-myelinated axons (purple), adjacent to a microglia/macrophage containing a dense lysosome and prominent rER (black and white arrows, respectively). The yellow arrow points to one of two adjacent mitochondria. Scale bar = 5 μm. (**G**) A microglia/macrophage extending a long process with dense lysosomes (black arrows). The cell on the *top right* has prominent mitochondria (yellow arrow) and rER (white arrow). Astrocyte processes (yellow) and pale amorphous material (green), likely reflecting axon loss, were present. Scale bar = 5 μm. (**H**) Structure resembling a swollen, neurofilament-rich axon (asterisk) close to the process of a microglia/macrophage (turquoise). Most axons appear normal but the pale areas of amorphous material (some shaded green) suggest axons have been lost. The pale nucleus probably belongs to an astrocyte. (**I**) Microglia/macrophage (turquoise) next to an organelle-rich axon swelling (purple) and astrocyte process (yellow). Scale bar = 2.5 μm. (**J**) A normal-appearing oligodendrocyte (pink) with a thin rim of cytoplasm is surrounded by myelinated axons. Scale bar = 2.5 μm. (**K**) Oligodendrocyte with an unusually large soma (as in **E** and **F**) containing mitochondria (yellow arrows) and swollen Golgi apparatus (turquoise arrow), suggesting its origin from the *Plp1*-tg mouse. Scale bar = 2.5 μm. (**L** and **M**) Microglia/macrophages apparently engulfing a degenerate myelin profile (**L**) and a cell resembling an oligodendrocyte (**M**). Scale bar = 5 μm. (**N**) Many normal-appearing myelinated axons alongside two redundant myelin profiles (black arrows). The cell in the *top right* has prominent rER, and a chromatin-rich nucleus. Scale bar = 5 μm. (**O**) Graph indicating total numbers of cell profiles (white bars) and numbers of ‘activated’ microglia/macrophages (grey bars) per area of interest (AOI) in untreated and transplant recipient P120 *Plp1*-tg mice. Bars represent mean ± SD. The transplant recipient harboured significantly fewer total cells (*P *<* *0.0001) including significantly fewer microglia/macrophages (*P *<* *0.0001) than the untreated control; taking each area of interest as an independent sample.

Finally, we asked if transplantation of neural precursors and restoration of wild-type myelin reduces neuroinflammation (Supplementary Fig. 2). In resin sections of the corpus callosum at P120, it was obvious at the level of the light microscope that there were fewer ‘angular’ cells with dark nuclei and cytoplasm in the transplant recipient compared with the untreated control (Fig. 8A–C). By electron microscopy, these dark, ‘angular’ cells contained dense chromatin, prominent rough ER and dense lysosomes typical of phagocytic microglia/macrophages (Fig. 8D–I). They were often observed close to degenerate myelin profiles (Fig. 8D and E), oligodendrocytes with swollen soma (Fig. 8F), astrocytic processes (Fig. 8G) or swollen axons (Fig. 8H andI). Their ‘angular’ appearance at the level of the light microscope was likely due to the fact they extended long processes (Fig. 8G). In contrast, in transplant recipients, it was more common to observe oligodendrocytes; some with a small perinuclear cytoplasm (Fig. 8J), presumably arising from the transplanted wild-type neural precursors; or with a large soma (Fig. 8K), presumably of *Plp1*-tg origin. In contrast to the untreated control, in which there were many areas filled with astrocyte processes (Fig. 8G, yellow) or devoid of normal cellular contents (Fig. 8G, green), presumably representing axon loss, the majority of the space was occupied by healthy-appearing myelinated and non-myelinated axons; although occasional redundant myelin profiles were observed (Fig. 8L). Activated microglia/macrophages were also occasionally observed, such as those illustrated in Fig. 8M and N, apparently engulfing a degenerate myelin profile (Fig. 8M) or an adjacent cell (Fig. 8N). Having identified these dense ‘angular’ cells as microglia/macrophages, we quantified total cell profiles or microglia/macrophages in resin sections. The total number of cells per area of interest (5866 μm^2^) and the number of microglia/macrophages were significantly decreased in the transplant recipient compared to the control (Fig. 8O). A similar reduction in microglia/macrophages was also observed by immunofluorescence in both transplant recipients terminated at P120.

## Discussion

Here we describe two elderly brothers with duplication of the *PLP1* locus and provide proof-of-principle, in a corresponding mouse model, for stem cell transplantation as a therapeutic approach to PMD caused by duplication mutations. In comparison to connatal forms of PMD, for which neural stem cell transplantation has undergone safety trial (Gupta *et al.*, 2019), the patients described here had relatively mild symptoms, which were nonetheless highly debilitating. Our patients’ symptom progression, radiological data and end stage pathology are compatible with relatively robust developmental myelination; a supposition supported by the fact that a cerebral biopsy from a 14-week-old relative revealed ‘normal myelin sheaths’ (Watanabe *et al.*, 1973). In contrast, connatal PMD is characterized by a marked lack, or complete absence, of myelin (van der Knaap and Valk, 1989; Duncan and Radcliff, 2016), raising the question whether cell transplantation is a viable option for classical forms of PMD, in which transplanted cells must compete with endogenous cells for axon coverage and neuron-derived trophic support (Barres *et al.*, 1992; Barres and Raff, 1994, 1999).

To our knowledge, with only one exception (Marteyn *et al.*, 2016), all previous experimental studies addressing cell transplantation as a therapy for PMD have been done in animal models of connatal PMD where there is a marked paucity of myelin from the outset (Duncan and Radcliff, 2016). Only the study of Marteyn *et al.* (2016) aimed at PMD caused by *PLP1* gene duplication, using our *Plp1*-tg mice (but the less severe line #66; Readhead *et al.*, 1994). However, these investigators used human progenitors as donor cells, which are well-known to be dominant, outgrowing mouse OPC in the chimeric brain (Windrem *et al.*, 2002). Thus, the current study was able to address the critical issue of how wild-type OPCs compete with *Plp1*-tg OPCs within the same species; a highly therapy-relevant question in humans. Indeed, we provide the first definitive demonstration using a transgenic model of PMD, where transplanted neural stem cells of the same species successfully compete against mutant OPCs and thus form myelin in the context of relatively normal developmental myelination by 'mutant' cells. This is likely relevant to other human leukodystrophies in which oligodendrocytes exhibit developmental defects but are nevertheless present as axon-ensheathing cells.

We previously demonstrated, using heterozygous female *Plp1* mutant mice that are mosaics harbouring two alleles of the X-linked *Plp1* gene, that despite being generated in equal numbers, a hierarchy of survival favours those oligodendrocytes that express the least deleterious *Plp1* allele. Thus, oligodendrocyte survival and myelination in a competitive environment is not determined solely by the nature of the mutation, but also by competing cells (Edgar *et al.*, 2002). We speculated this was due to the ‘dominant’ cell being better able to associate with axons and obtain essential survival factors. In the current study, we found that despite *Plp1*-tg nOPCs extending processes to a similar extent as their wild-type counterparts *in vitro*, transplanted wild-type neural precursors eventually produced large amounts of compact myelin in *Plp1*-tg transplant recipients. This suggests that the exogenous wild-type cells (with a competitive advantage) outcompete the endogenous a*Plp1*-tg OPCs to replace *Plp1*-tg myelin as it degraded.

Incidentally, we also observed that the capacity of wild-type aOPCs to extend processes *in vitro* is reduced in comparison to that of nOPCs (Fig. 7C and D). This, to our knowledge, is the first report to show this phenotypic difference between nOPCs and aOPCs and has relevance for understanding failure of remyelination, which is impaired with increasing age (Neumann *et al.*, 2019), in multiple sclerosis.

The pathology observed in our patients is characterized by regions containing large numbers of appropriately myelinated axons interspersed with naked and thinly sheathed axons and areas of axonal loss. While some of the naked and thinly sheathed axons likely reflect persistent dysmyelination, others clearly represent demyelination. This conclusion was also drawn in another recent report describing findings in the corpus callosum in the same patients (Laukka *et al.*, 2016). In fact, our patients exhibited a phenotype resembling that in the #72 *Plp1*-tg line described in this study and in other lines with intermediate or low *Plp1*/*PLP1* transgene dosage, that undergo demyelination and axonal degeneration in adulthood (Mastronardi *et al.*, 1993; Inoue* et al.,* 1996; Anderson *et al.*, 1998, 1999).

In the patients, axonal degeneration was observed in all regions examined but particularly in the periphery of the lateral and in the posterior columns of the spinal cord. In contrast, in patients lacking PLP1 due to a null mutation of the gene, axon degeneration primarily affects the corticospinal tracts and fasciculus gracilis, consistent with length-dependent degeneration (Garbern *et al.*, 2002). The current findings contribute to evidence that axonal degeneration is a major feature of some phenotypes of PMD (Garbern *et al.*, 2002; Laukka *et al.*, 2016); however, the mechanisms remain obscure. Experimental studies in line #66 *Plp1*-overexpressing mouse model of *PLP1* duplication, demonstrate that CD8-positive T cells contribute to the pathogenesis of myelin and axon injury (Ip *et al.*, 2006) and *Plp1* ‘overexpression’ in mice is clearly associated with a marked neuroinflammation involving microglia/macrophages, astrocytes and limited T-cell infiltration (Ip *et al.*, 2006, 2007; Edgar *et al.*, 2010).

However, the earliest axon changes in mouse models and in the patients described here likely represent a secondary response to the injured ‘mutant’ myelin. Whilst the level of CNP in the biopsy specimen from the 14-week-old relative of our patients was reported as normal (Watanabe *et al.*, 1973), we found reduced levels of CNP and also of MAG in our patients. In contrast, MBP was present in myelin at levels similar to controls. While an increase in *PLP1*/DM20 was anticipated, it is not clear why CNP and MAG were reduced although a reduction in both has also been noted in hemi- and homozygous line #66 *Plp1*-tg mice (Karim *et al.*, 2007). CNP acts as an intracellular strut to prevent compaction of the cytoplasm-filled space at the periphery of the oligodendrocyte process (Snaidero *et al.*, 2017) and reduced levels of CNP could potentially interfere with trafficking between the glial cell body and the glia-axonal junction, resulting in impaired metabolic support of axons (Saab *et al.*, 2013; Stassart *et al.*, 2018) and secondary axon degeneration (Lappe-Siefke *et al.*, 2003; Edgar *et al.*, 2009). Reduction in levels of MAG, which binds to receptors on the axolemma (Yang *et al.*, 1996; Yamashita *et al.*, 2002; Pan *et al.*, 2005; Schnaar and Lopez, 2009), could also potentially interfere with the normal glial-axonal interaction. In rodents with spontaneous mutations in myelin genes, loss of MAG from the oligodendrocyte inner tongue is associated with demyelination (Bö *et al.*, 1995; O’Connor *et al.*, 2000; Song *et al.*, 2001) and degeneration of the myelin inner tongue has been reported in aged hemizygous #66 *Plp1-*tg mice (Fig. 8; Anderson *et al.*, 1998). Cell transplantation appeared to reduce axon loss in this study (compare Fig. 8I and N), but whether this was due directly to restoration of normal myelin and of myelin proteins, to reducing neuroinflammation including T-cell infiltration, or a combination of both, remains undetermined.

Two previous reports of patients with confirmed *PLP1* duplication (Cremers *et al.*, 1987; Harding *et al.*, 1995) emphasize the myelin deficit. In contrast to the brothers described here, both patients were young at the time of death. The patient described by Harding *et al.* (1995) likely has a triplication of the *PLP1* gene, and the patient described by Cremers *et al.* (1987) had an interstitial translocation of the X chromosome, which may account for the more complex syndrome including dysmorphic features not generally observed in patients with classical PMD. A similarly severe phenotype in mice with high *Plp1*/*PLP1* transgene dosage (Kagawa *et al.*, 1994; Readhead *et al.*, 1994; Anderson *et al.*, 1999) is associated with sequestration of *PLP1* and cholesterol in endosomes/lysosomes (Simons *et al.*, 2002) that, paradoxically, can be rescued by cholesterol supplementation in a mouse model (Saher *et al.*, 2012), but not in PMD patients, likely due to differences in blood–brain barrier permeability between the two species in the context of this disease (Stumpf *et al.*, 2019). However, a ketogenic diet, which does not require a permeable blood–brain barrier for entry of ketones into the CNS, improves axon function and restores axonal mitochondrial size in the same mouse model (Stumpf *et al.*, 2019). This raises the possibility that early dietary intervention combined with transplantation of neural precursors could markedly improve disease prognosis through restoration of myelin and dietary-mediated neuroprotection, currently a critical unmet clinical need in demyelinating disease (Franklin and Kotter, 2008; Irvine and Blakemore, 2008; Wilkins and Scolding, 2008).

## Supplementary Material

awaa080_Supplementary_Data

## Abbreviations

a/nOPC =: adult/neonatal oligodendrocyte precursor cell
PMD =: Pelizaeus-Merzbacher disease
