# Supplementary material for: Neural stem cells restore myelin in a demyelinating model of Pelizaeus-Merzbacher disease
Source: Brain. 2020 May 18;143(5):1383–99. doi: 10.1093/brain/awaa080 (PMC7462093; doi:10.1093/brain/awaa080)
Supplement: awaa080_Supplementary_Data [file awaa080_supplementary_data.zip › awaa080-suppl_data/brain-2019-01726-File010.pdf]

Supplementary Materials and Methods

Animals

Animals were maintained in the Biological Services Facility at the University of Glasgow in a 12 hour light/dark cycle at RT 19-21 ^{o}C. They were housed with sex-matched littermates in conventional caging, up to 6 animals per cage and provided with food and water ad libitum. In general, only stud males were housed alone, to prevent fighting.

Whenever possible, littermates of the two genotypes (wild type and *Plp1*-tg) were used for quantification of cells and myelinated axons, but if both genotypes were not present in the same litter, comparisons were made between animals from different litters, which were all part of the same inbred colony.

In general, tissue from 3 *Plp1*-tg and 3 wild type mice were assessed at each of 4 ages. These came from at least two individual litters, for each time point. Thus, mice from at least 8 litters were examined by electron microscopy and mice from at least 8 litters were examined by light microscopy. One pair of mice (wild type and age matched *Plp*-tg littermate or colonymate) was considered n=1 for the purpose of statistical analyses. Observers were blinded to the phenotype of young animals which were usually indistinguishable, in terms of cage behaviour, from controls. However, *Plp1*-tg mice displayed a phenotype from P90 onwards that made them easily distinguishable from wild type. This was also the case for tissue from *Plp1*-tg mice from P60 onwards, thus the experimenter was not generally ‘blinded’ to the genotypes during analysis of tissue or inspection/selection of animals. Nonetheless, the results are in line with the observations from other reports [Edgar *et al.* 2010;Ruest *et al.* 2011]. Cage behaviour was assessed by the first author who is a trained veterinarian. As disturbing the animals increased the likelihood of seizures, cages were

moved gently for inspection of animals' cage behaviour and direct handling was avoided. Animals nearing P120 (i.e. fromP100) were monitored at least daily for signs of prolonged seizure activity.

Transplantation into the adult corpus callosum

Volatile anaesthetic was used because it is easy to regulate the dose and avoid overdose. Anaesthesia was induced in homozygous *Plp1*-tg mice (age P100) with 5% isoflurane and maintained with 2% isoflurane, mixed with 30% O_2 and 70% NO_2. Deeply anaesthetised animals were fixed in a rodent stereotactic frame with a mouse face mask and non-traumatic ear bars. Presurgical subcutaneous injection of 0.3ml carprofen (Rimadyl® large animal injection solution, Pfizer; diluted in 1:100 sterile H_2O to a concentration of 0.5 mg/ml) was administered. Right sided craniectomies were performed, exposing the right lateral cerebral hemisphere and the intact dorsal sagittal sinus. A glass capillary, connected to a microinfusion pump (CellTram Oil manual piston pump, Eppendorf) containing 2 µl of a dense neurosphere suspension was placed according to the stereotactic coordinates (Paxinos and Franklin, 2001). The injection sites were -0.58 mm caudal to Bregma point 0, 0.5 mm lateral to the midline suture and 1.5 mm ventrally to the brain surface and 1.34 mm caudal to Bregma point 0, 0.5 mm lateral to the midline suture and 1.5 mm ventrally from the brain surface. At each, site the glass capillary was advanced for a further 0.5 mm, left in place for 1 minute and then retracted, to counteract any compression of the brain tissue during insertion of the capillary. A 1µl cell suspension was injected at a rate of 0.1µl/min. Before recovering the mouse from anaesthesia, a subcutaneous injection of 0.5 ml physiological saline was given to prevent dehydration. For continuous pain management, Rimadyl® was added to the drinking water to a concentration of 0.05 mg/ml for three days.

Transplantation in neonates

The entire litter was removed from the mother prior to surgery to minimise the risk of rejection afterwards, and the mother was transferred to a clean cage lined with a cotton pad. Pups were anaesthetised with 5% isoflurane and 100% O_2 and transferred, one at a time, onto a sterile cotton pad on top of a 37°C heating pad. The primary operator fixed the head by holding it gently and a total of 4 injections were made into the rostral part of the cerebrum, immediately right and left of Bregma point 0, and into the caudal part of the cerebrum, immediately right and left of where the midline suture crosses the caudal suture line. Cells were injected using a tapered 30 G needle attached to a 5 μl Hamilton syringe (Hamilton 65RN). The needle (which was marked with an indelible pen) was inserted by the primary operator to a depth of 1.5 mm, and 0.5 μl cell suspension was injected slowly by a 2nd operator into each site under an operating microscope. After injection, the pups were left to recover in a heated recovery box and the entire litter was returned simultaneously to the mother ~10 minutes after the final injection.

Preparation of OPCs from adults and neonates

Adult mice were killed humanely in slowly increasing levels of CO_2 and the brains were removed and placed into ice cold Leibovitz's L15 (L15, Gibco, 21083-027). Dissected corpus callosa were cut into 1 mm^3 pieces and incubated in 3 ml HBSS (Sigma, H-4891), 0.125% trypsin (Sigma, T-3924) 30 µl of DNAse (50mg/ml, Sigma, DN25-100mg) for 90 minutes in 5% CO_2 at 37°C. Postnatal day 5 pups were killed by decapitation following anaesthesia in isofluorane and the brains dissected and cut into 1 mm^3 pieces and incubated as above.

Heat inactivated foetal bovine serum (HIFBS) inL15 (10 % final) was added to stop the digestion, tissue was centrifuged at 1000 rpm for 10 minutes in a benchtop centrifuge and the

pellet was resuspended in 2 ml ice chilled 10% HIFBS/L15 and triturated 6 times through a flame-polished glass pipette. The cell suspension was diluted to a total volume of 6.5 ml using 10 % HIFBS in L15 with 65 µl of DNAse (50 mg/ml). A 90 % Percoll® (Sigma, 4937) solution was obtained with 10x D-PBS (Gibco, 14200-067). 3 ml 90% Percoll® was added to the cell suspension, gently mixed then centrifuged in an Oakridge tube at 30000 g for 30 minutes at 4°C. OPCs were harvested from immediately above the red blood cell layer, diluted in 5 ml 10% HIFBS in L15 and centrifuged at 1000 rpm for on a benchtop centrifuge for10 minutes RT. The pellet was resuspended in 100 µl 10 % HIFBS/L15 (adult cells) or 1 ml 10 % HIFBS/L15 (neonatal cells) for quantification using a haemocytometer then diluted to a final concentration of 10^6 cells/ml or 3-4 x 10^5 cells/ml, respectively. Wild type cells expressing GFP and *Plp1*-tg cells were mixed together in equal numbers and a volume of 100 µl of cell suspension in 10 % HIFBS/L15 was pipetted onto a poly-L-lysine coated 13 mm diameter glass coverslip (3 coverslips per 35 mm Petri-dish) and after the cells adhered (≥ 2 hours) the volume per Petri-dish was made to 1 ml with 700 µl differentiation medium comprising DMEM (Gibco 31885-023) containing Penicillin/Streptomycin, 30% D-glucose (to 4500 mg/l final concentration), 10 ng/ml biotin, 50 nM hydrocortisone, 10 µg/ml insulin, 0.5% hormone mix (stock concentration 1 mg/ml apo-transferrin, 20 mM putrescine, 4 µM progesterone, and 6 µM selenium. Cells were incubated at 37°C in 5% CO_2, then fed three times a week by replacing half the medium with serum-free differentiation media.

Antibodies and immunohistochemistry on mouse tissue

Antibodies were anti-MBP (1:500, as for human study); anti-CD3 (MCA1477T; 1:100), anti-CD45 (MCA1388; 1:600) and anti-CD169 (MCA884; 1:100) (all Serotec, raised in rat); anti-caspase 3 (CST #9661; 1:5000) anti-NG2 (Millipore AB5320; 1:500) and -anti GFP (Abcam Ab290; 1:1000), anti-PLP/DM20 (1:600; as above), anti-PDGFRα (1:500)(all raised in

rabbit); anti-CC1 (Abcam Ab16794; 1:100); anti-BrdU (Sigma-Aldrich B8434; 1:500); anti-GFAP (Sigma-Aldrich G3893; 1:1000); and anti-OLIG1 (Millipore MAB5540; 1:1000), SMI32-R; (1:1500) (all raised in mouse). Secondary antibodies labelled with fluorescein isothiocyanate (FITC, Cambridge Bioscience; 1:100) Alexa Fluor 488 (Invitrogen; 1:1000) or sulforhodamine 101 acid chloride (Texas Red, TxR, Cambridge Bioscience; 1:100) were used. 4',6-diamidino-2-phenylindol (DAPI, Sigma) was used to counterstain cell nuclei.

In all cases except for OLIG1, BrdU and CC1, sections were permeabilised in -20°C methanol (Fisher Scientific), blocked in 10% normal goat serum (NGS, Sigma-Aldrich) in PBS. Primary antibodies were applied overnight at 4°C and secondary antibodies for ~1 h RT, both in blocking solution. DAPI (1:1000 in PBS) was applied for one minute.

For double labelling with anti-BrdU, anti-PDGFRα was applied as above then tissue was immersed in 50% ethanol/50% glacial acetic acid and then in 50% HCl 1% Triton-X 100, both 10 minutes. Anti-BrdU in 1% Triton-X 100 and 10% NGS was applied overnight at 4°C. Secondaries were applied for ~2 h RT.

For anti-OLIG1 or anti-CC1, sections were simultaneously blocked and permeabilised in 0.5% Triton-X 100 in 10% NGS/PBS or 0.1% Triton-X 100 and 0.2% porcine skin gelatine in PBS, respectively for 1 h. Primary and secondary antibodies were applies as above in the respective blocking solutions.

β - galactosidase staining

Following perfusion fixation with 4% PFA, brains were dissected and 1 mm thick transverse slices were obtained using a brain matrix (Harvard Apparatus). Samples were post fixed for

10 minutes on ice in 4% PFA, washed twice for 10 minutes on ice in fresh 2 mM $MgCl_2$/PBS then 10 minutes in 1 mM $MgCl_2$, 0.01% sodium deoxycholate, 0.02% NP40 (both Sigma-Aldrich) and immersed for 3 h RT with X-gal staining solution (20 mM $K_3Fe(CN)_6$, 20 mM $K_4Fe(CN)_6$ $3H_2O$, 2 mM MgCl2, 0.01% sodium deoxycholate, 0.02% NP40) and X-gal 1 mg/ml in PBS, pH 7.4. Finally, samples were washed in PBS and in water.

Reference List

Edgar JM, McCulloch MC, Montague P *et al*. Demyelination and axonal preservation in a transgenic mouse model of Pelizaeus-Merzbacher disease. EMBO Mol Med 2010; 2: 42-50.

Ruest T, Holmes WM, Barrie JA *et al*. High-resolution diffusion tensor imaging of fixed brain in a mouse model of Pelizaeus-Merzbacher disease: comparison with quantitative measures of white matter pathology. NMR Biomed 2011; 24: 1369-1379.

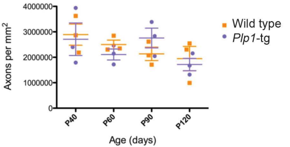

Axon density in the corpus callosum is similar in both genotypes

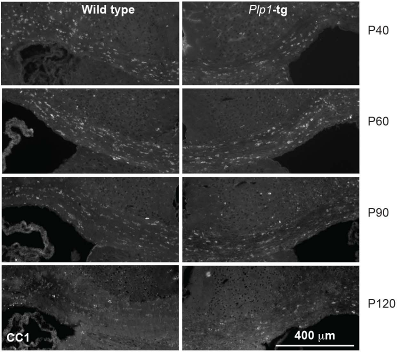

CC1 +ve oligodendrocytes in the corpus callosum of WT and tg

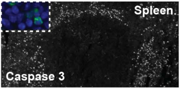

Spleen, stained in parallel with brain tissue, acts as a positive control for caspase 3

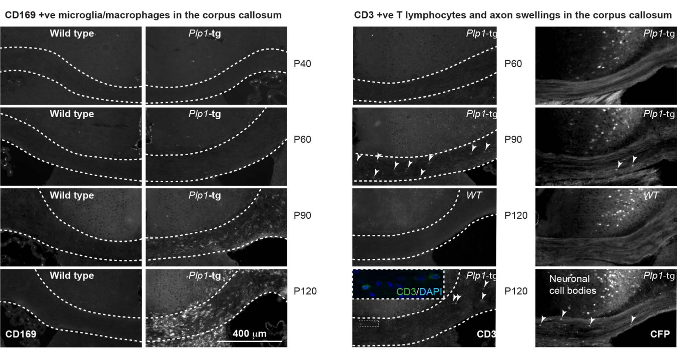

CD169 +ve microglia/macrophages in the corpus callosum
CD3 +ve T lymphocytes and axon swellings in the corpus callosum
Wild type
Plp1-tg
P40
P60
P90
P120
WT
CD3/DAPI
Neuronal cell bodies
CD169
400 μm
CD3
CFP

Supplementary Table 1. Quantitative RT-PCR analysis of RNA from brain tissue from patient 2 and a multiple sclerosis patient.

PLP+DM20 (exon 1 to exon 3)

	Exon 1 to exon 3	Normalized to GAPDH	Relative to MS
Duplication	640743*	1.38	1.25
MS	673335	1.11	1.00

* Arbitrary densitometric units
