# Supplementary figures and images for: Neural stem cells restore myelin in a demyelinating model of Pelizaeus-Merzbacher disease
Source: Brain. 2020 May 18;143(5):1383–99. doi: 10.1093/brain/awaa080 (PMC7462093; doi:10.1093/brain/awaa080)

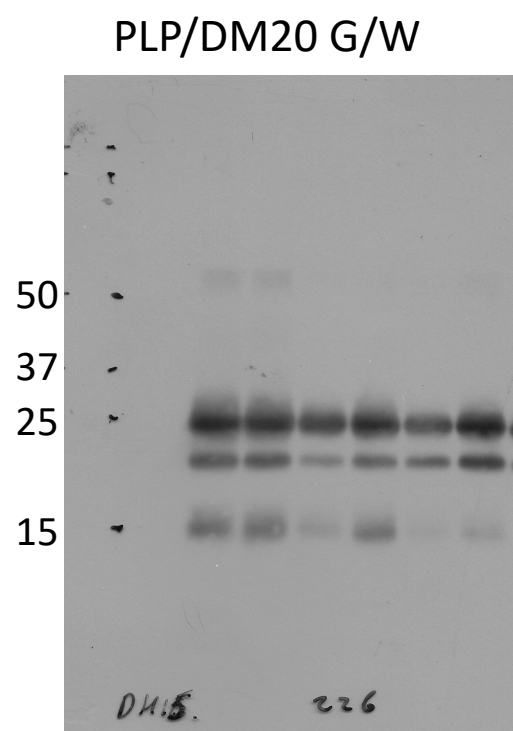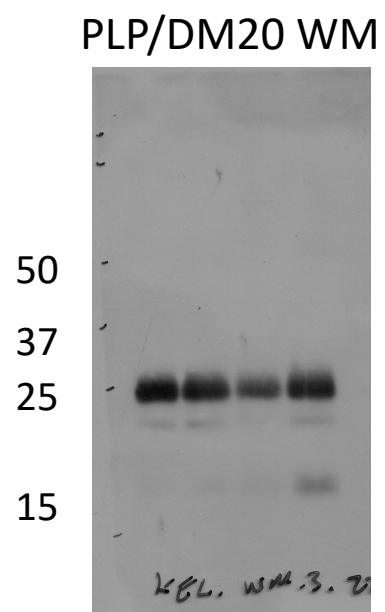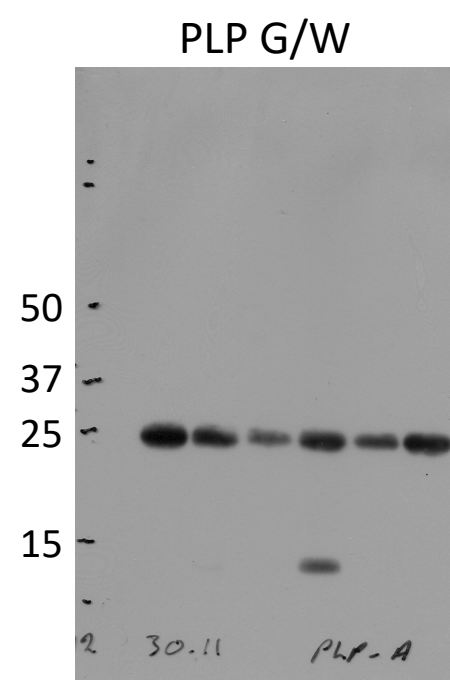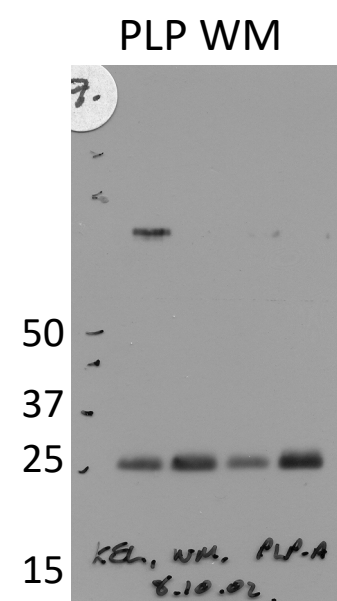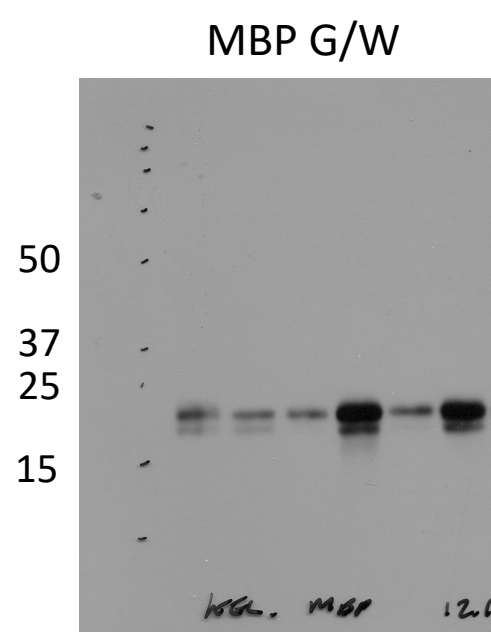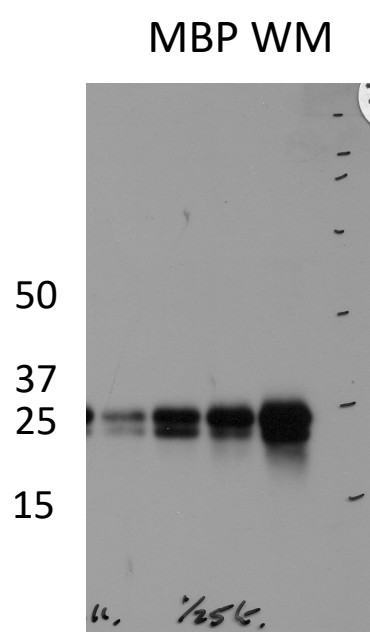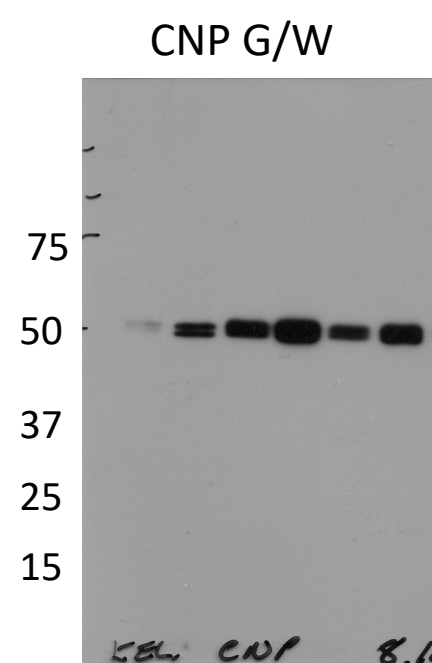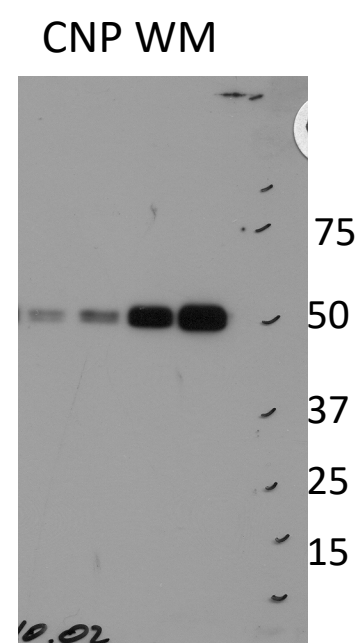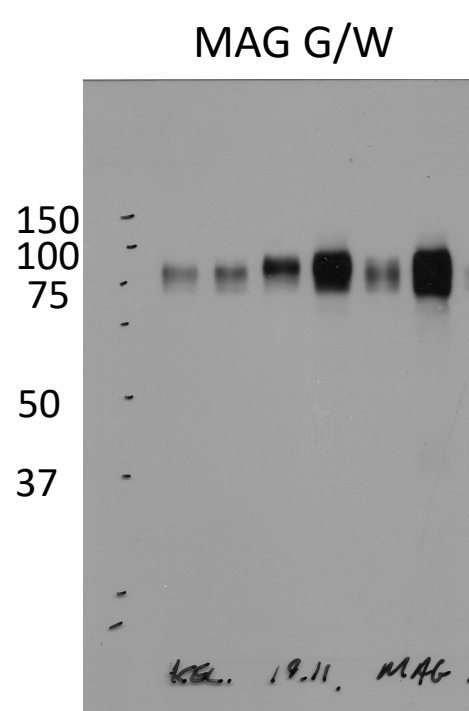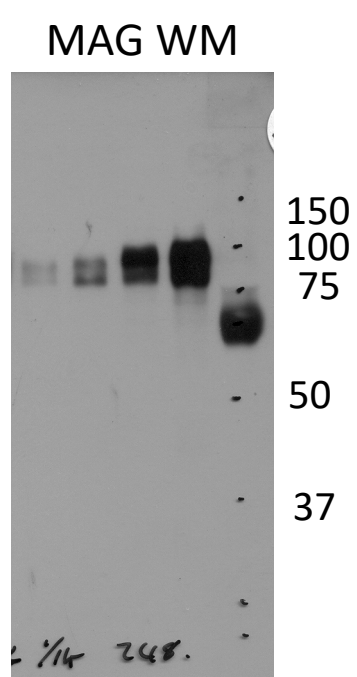

Supplement: awaa080_Supplementary_Data [file awaa080_supplementary_data.zip › awaa080-suppl_data/brain-2019-01726-File012.pdf]
